# Evaluation of Prothrombin Time and Activated Partial Thromboplastin Time in Hypertensive Patients Attending a Tertiary Hospital in Calabar, Nigeria

**DOI:** 10.1155/2014/932039

**Published:** 2014-11-16

**Authors:** Nnamani Nnenna Adaeze, Anthony Uchenna Emeribe, Idris Abdullahi Nasiru, Adamu Babayo, Emmanuel K. Uko

**Affiliations:** ^1^Department of Medical Laboratory Science (Haematology and Blood Group Serology Unit), University of Calabar, PMB 1115, Calabar, Cross River State, Nigeria; ^2^Department of Chemical Pathology, University of Abuja Teaching Hospital, PMB 228, Gwagwalada, Abuja, Nigeria; ^3^Department of Medical Microbiology, University of Abuja Teaching Hospital, PMB 228, Gwagwalada, Abuja, Nigeria; ^4^Department of Medical Laboratory Science, University of Maiduguri, PMB 1069, Maiduguri, Nigeria

## Abstract

*Introduction*. Several biomedical findings have established the effects of hypertension on haemostasis and roles of blood coagulation products in the clinical course of hypertension.* Methods*. This cross-sectional study aimed at determining effects of hypertension on prothrombin time (PT) and activated partial thromboplastin time (APTT) in hypertensive patients in comparison with normotensive subjects attending a tertiary hospital in Calabar. Forty-two (42) hypertensive patients and thirty-nine (39) normotensive control subjects were investigated for PT and APTT using Quick one-stage methods.* Results*. Systolic blood pressure (SBP) and diastolic blood pressure (DBP) correlated positively with APTT (*r* = 0.3072, *r* = 0.4988; *P* < 0.05) in hypertensive patients. DBP, SBP, PT, and APTT were significantly higher in hypertensive patients when compared to normotensive subjects (*P* < 0.05). DBP correlated negatively with duration of illness (*r* = −0.3097; *P* < 0.05) in hypertensive patients and positively with age of normotensive subjects (*r* = 0.3523; *P* < 0.05).* Conclusion*. The results obtained indicated that measurements of PT and APTT may serve as indices for evaluating hemostatic abnormalities in hypertensive patients and guide for antihypertensive therapy. However, to have better understanding of hemostatic activities in hypertension, it is recommended to conduct D-dimer, platelet factors, and protein assays.

## 1. Introduction

Cardiovascular diseases account for about one-third of premature deaths in men and one-quarter in women and arterial hypertension is one of the most significant risk factors for cardiovascular diseases [1]. Despite current knowledge, extensive clinical and experimental research, the cause of hypertension remains unknown in about 95% of all cases [1]. Hypertension is a chronic medical condition in which the blood pressure is indisputably elevated (≥140/90 mmHg). It is one of the most common worldwide diseases afflicting humans. Globally cardiovascular disease accounts for approximately 17 million deaths a year, nearly one-third of the total. Of these, complications of hypertension account for 9.4 million deaths worldwide every year. Hypertension is responsible for at least 45% of deaths due to heart disease [2]. Due to several associated morbidity, mortality, and economic cost to society, hypertension is now a serious public health challenge to both developed and developing nations [3, 4].

According to recent epidemiologic data, the overall prevalence of 28% of the general population is suffering from hypertension in Sub-Saharan Africa, the prevalence rate in Nigeria is put at 22% with steady annual increase [5].

Hypertension is classified as either primary or secondary. It is primary when no medical cause can be found to explain the raised blood pressure. This type represents between 90 and 95% of hypertension cases [6]. Secondary hypertension represents approximately 10% of all hypertension cases. Identifiable underlying causes of secondary hypertension are kidney disease, renal hyperaldosteronism, and pheochromocytoma. Secondary hypertension has specific therapy; it is potentially curable and often distinguishable from primary one on clinical grounds [7].

Risk factors associated with hypertension include age, race, family history, obesity, sedentary lifestyle, alcohol abuse, and stress among others. Hypertension increases hardening of the arteries and predisposes individuals to heart disease, peripheral vascular disease, and strokes [7–10].

Hypertensive patients are at high risk for the development of cardiovascular diseases [11], whereas several studies have shown that treatment of hypertension diminishes the prevalence of cardiovascular diseases [12–14]. The existence of hypertension, especially in combination with other risk factors, is disadvantageous for the prognosis of cardiovascular diseases [15]. The integrity of the blood vessels is essential because damage of the intima, which may occur in hypertension, can finally cause atherosclerosis. This kind of patient is likely to develop increased platelet aggregation with heart and blood vessel problems as possible sequelae [16]. Moreover, blood vessel damage activates the coagulation system, which may also stimulate the progress of atherosclerosis.

Coagulation abnormalities in pregnant women have been reported to be more serious in women with hypertension (preeclampsia) than in those without hypertension [17]. In patients with borderline hypertension, even before the appearance of clinical manifestations of vascular damages [18], coagulation activation seems to be already present [18].

Besides platelet aggregability and coagulation activation, fibrinolysis, that is, plasma tissue-type plasminogen activator activity, appears to be a major factor related to the risk of cardiovascular disease [19–22].

Prothrombin time (PT) and activated partial thromboplastin time (APTT) have been shown to be associated with elevated systolic and diastolic blood pressures in hypertensive and normotensive patients [23]. In a study which involved the inclusion of one hundred and one patients with hypertension of mild to moderate grades, attending Al-Najaf Teaching Hospital, Iraq, significant differences were found in PT and APTT between hypertensive and normotensive patients [24]. The prothrombin time (PT) and its derived measures of prothrombin ratio (PR) and international normalized ratio (INR) are measures of the extrinsic pathway of coagulation. The APTT in contrast to the PT measures the activity of the intrinsic pathways of coagulation. Endothelial damage, platelet hyperactivity, and other changes of blood coagulation may play a role in the vascular complications of essential hypertension [25–27].

The main aim of this study is to assess possible association between prothrombin time and activated partial thromboplastin time with effects of blood pressures in hypertensive and normotensive subjects and their significant utility for screening hemostatic dysfunction in hypertensive patients.

## 2. Materials and Methods

### 2.1. Study Area

The study was conducted at the University of Calabar Teaching Hospital, Calabar, Cross River State. Calabar is the capital city of Cross River State which is divided into Calabar Municipal and Calabar South Local Governments and is located at the coastal southeastern area (4°57′N 8°19′E) of Nigeria. It has an area of 604 km^2^ and a population of 371,022 at the 2006 census.

### 2.2. Study Population

A total of eighty-onesubjects were enrolled into the study. Forty-two (42) hypertensive patients aged between 30 and 80 years and thirty-nine (39) normotensive control subjects were investigated for PT and APTT using Quick one-stage methods. The selection of patients was done with the support of the physician and nursing staff of the Medical Outpatient Department (MOPD) of University of Calabar Teaching Hospital (UCTH), Calabar. The procedures employed consisted of a questionnaire interview, taking of patient's history, and blood pressure measurement.

### 2.3. Inclusion Criteria for Hypertensive Patients

The inclusion criteria are as follows:

- the presence of the history of primary hypertension;
- elevated blood pressure (≥140/90 mmHg);
- no history of administration of anticoagulant therapy;
- no history of chronic viral infection and/or liver diseases (HBV, HCV, HIV, and alcohol consumption);
- having not been on long-term drug regimen;
- must be within normal range body mass index;
- being with no trace of underlying causes.

### 2.4. Exclusion Criteria for Hypertensive Patients

The exclusion criteria are as follows:

- the absence of family history of primary hypertension;
- normal blood pressure (<140/90 mmHg);
- those on anticoagulant therapy;
- those with chronic viral infections and/or liver diseases such as HBV, HCV, HIV, and alcoholism;
- obese individuals.

### 2.5. Inclusion Criteria for Normotensive Control Subjects

The inclusion criteria are as follows:

- the absence of family history of hypertension;
- normal blood pressure (<140/90 mmHg);
- no history of administration of anticoagulant therapy;
- no history of chronic viral infection and/or liver diseases (HBV, HCV, HIV, and alcohol consumption);
- having not been on long-term drug regimen;
- must be within normal body mass index;
- with no trace of underlying causes.

### 2.6. Exclusion Criteria for Normotensive Control Subjects

The exclusion criteria are as follows:

- the presence of family history of hypertension;
- elevated blood pressure (≥140/90 mmHg);
- the absence of family history of primary hypertension;
- normal blood pressure (<140/90 mmHg);
- those on anticoagulant therapy;
- those with chronic viral infections and/or liver diseases such as HBV, HCV, HIV, and alcoholism;
- obese individuals.

### 2.7. Ethical Clearance and Informed Consent

The ethical clearance was obtained from the Ethical Committee of UCTH, Calabar, Cross River State, Nigeria. Informed consent was obtained from all participating subjects. This was done via an informed consent form duly completed by all the subjects.

### 2.8. Questionnaire

Semistructured administered questionnaires were used to obtain data such as age, marital status, clinical signs and symptoms, and provisional diagnosis.

### 2.9. Sample Collection

Four and a half-millilitres (4.5 mL) of blood sample was drawn from hypertensive and normotensive subjects and discharged into a 0.5 mL of 3.13% trisodium citrate sample bottle. The anticoagulated samples were centrifuged at 4000 rpm for 10 minutes and platelet poor plasma stored in a deep freezer at −20°C until assayed. Standard method of Quick one-stage analysis by [28] was used for PT and APTT.

### 2.10. Statistical Method

The generated data was systematically analysed as appropriate for means, standard deviation, Student's *t*-test, Pearson's correlation analysis, and analysis of variance on Microsoft Excel and SPSS software version 18 (California Inc.). Results were presented as the mean ± standard deviation. A two-sided *P* < 0.05 was considered statistically significant for *t*-test (used to determine the differences between the groups) and Pearson's correlation analysis was used to determine the intervariable associations of the various groups.

## 3. Results

Table 1 shows the mean and standard deviation for the systolic pressure, diastolic pressure, prothrombin time test, and activated partial thromboplastin time test of hypertensives and normotensives including the duration of hypertensive subjects enrolled in the study. The mean and standard deviation for systolic blood pressure, diastolic blood pressure, prothrombin time test, activated partial thromboplastin time test, and duration of hypertensives were 157.02 ± 18.78 mmHg, 95.12 ± 13.00 mmHg, 14.45 ± 1.97 seconds, 35.43 ± 5.05 seconds, and 4.06 ± 5.93 years, respectively, while the mean and standard deviation for the systolic pressure, diastolic pressure, prothrombin time test, and activated partial thromboplastin time test of normotensives studied were 117.67 ± 11.65 mmHg, 79.17 ± 10.99 mmHg, 13.60 ± 1.19 seconds, and 32.57 ± 3.23 seconds, respectively.

Diastolic blood pressures, systolic blood pressures, Prothrombin Time Test and Activated Partial Thromboplastin Time Test were significantly higher in hypertensive patients when compared to normotensives (*P* < 0.05).

Based on the duration of hypertension, the hypertensive patients were grouped into three different groups (≤3 years, 4–7 years, and ≥8 years); their prothrombin time was 14.28 ± 1.80, 15.00 ± 2.67, and 14.50 ± 1.87 seconds, respectively, while their activated partial thromboplastin time was 34.89 ± 4.57, 37.37 ± 6.07, and 35.33 ± 6.05 seconds, respectively, as shown in Table 2.

**Figure 1:**
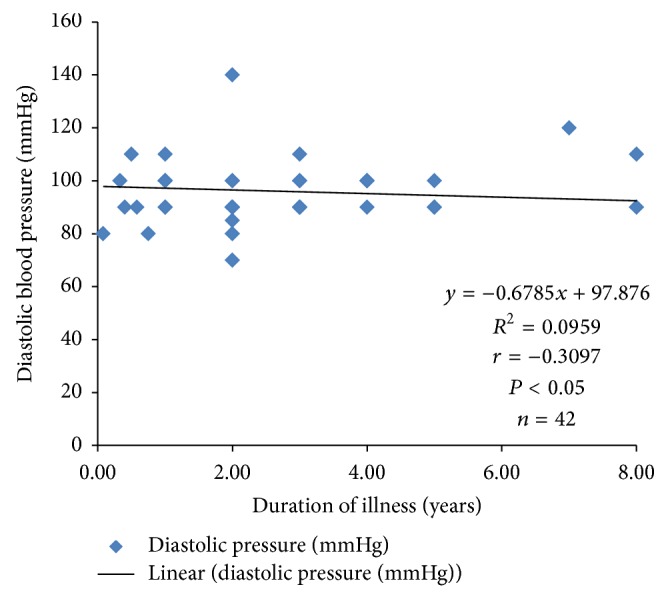
Correlation plot of diastolic blood pressure against duration of illness in hypertensive patients.

**Figure 2:**
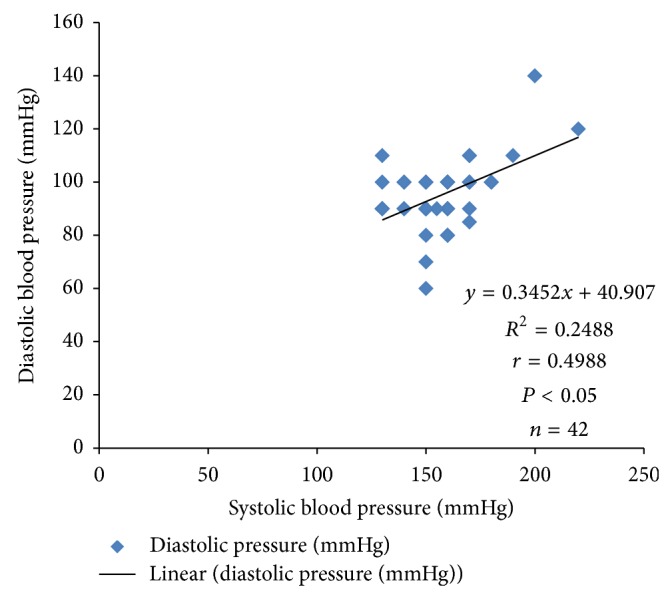
Correlation plot of systolic blood pressure against diastolic blood pressure in hypertensive patients.

**Figure 3:**
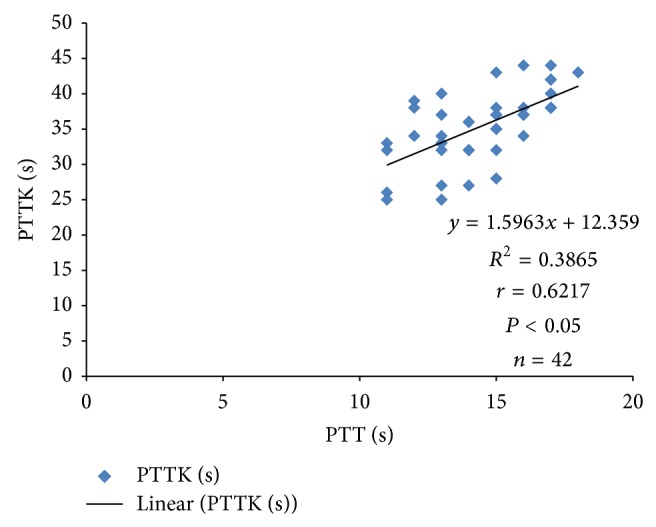
Correlation plot of PTTK against PTT in hypertensive patients.

**Figure 4:**
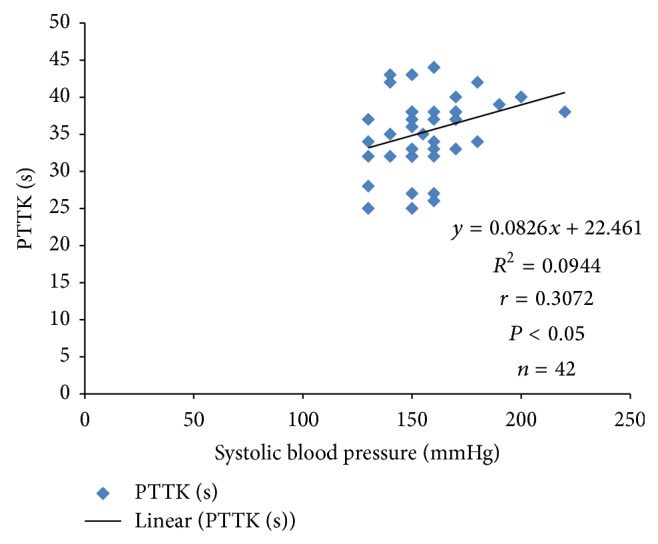
Correlation plot of PTTK against systolic blood pressure in hypertensive patients.

**Figure 5:**
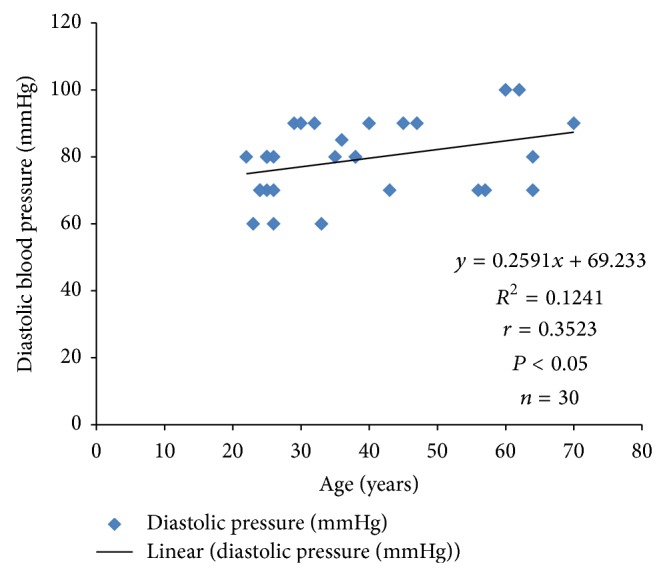
Correlation plot of diastolic blood pressure against age in normotensive subjects.

**Figure 6:**
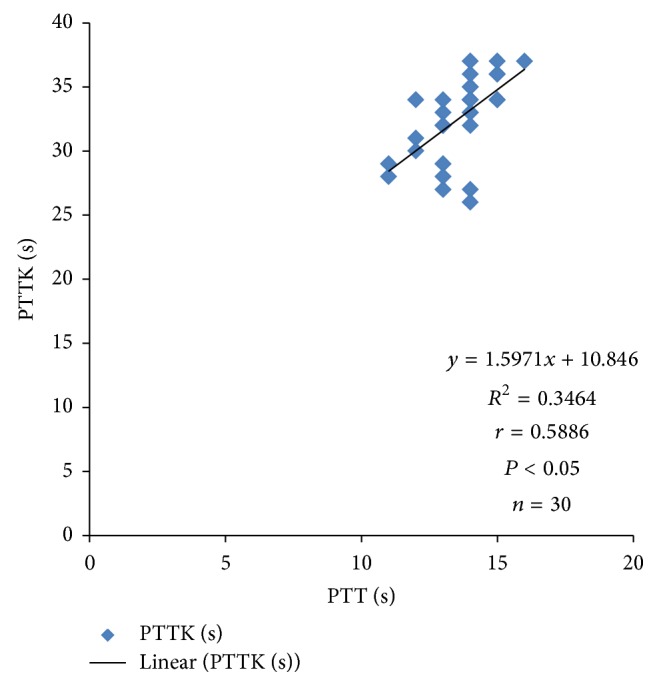
Correlation plot of PTTK against PTT in normotensive subjects.

**Figure 7:**
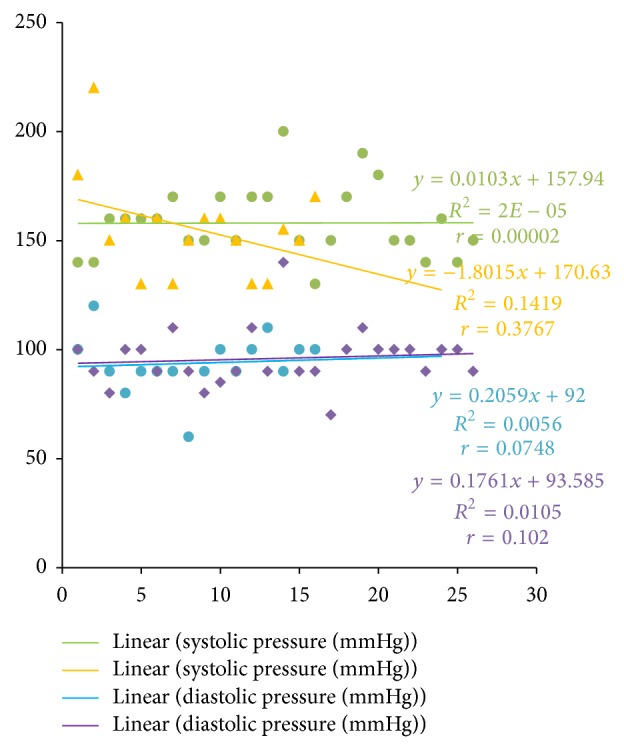
Correlation of systolic and diastolic blood pressure of hypertensive subjects across gender distribution. Purple lines and points: females. Blue line and points: males. Green line and points: females. Yellow line and points: males. Number of male hypertensive subjects (*n*) = 16. Number of female hypertensive subjects (*n*) = 26.

**Figure 8:**
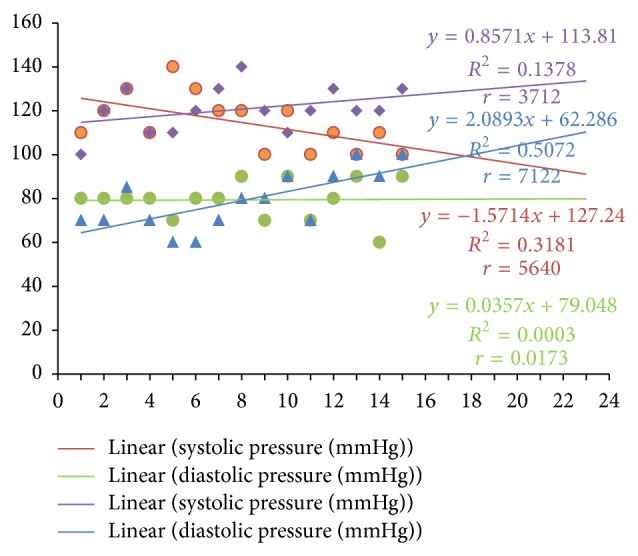
Correlation of systolic and diastolic blood pressure of normotensive subjects across gender distribution. Purple lines and points: females. Blue line and points: females. Green line and points: males. Red line and points: males. Number of male normotensive subjects (*n*) = 15. Number of female normotensive subjects (*n*) = 15.

Systolic blood pressure correlated positively with PTTK (*r* = 0.3072; *P* < 0.05) (Figure 4) and with diastolic blood pressure (*r* = 0.4988; *P* < 0.05) (Figure 2) in hypertensive patients, respectively. Diastolic blood pressure correlated negatively with duration of illness (*r* = −0.3097; *P* < 0.05) in hypertensive patients (Figure 1) and positively with age (*r* = 0.3523; *P* < 0.05) in normotensive subjects (Figure 5). A positive correlation was observed between PTT and PTTK both in hypertensive patients (*r* = 0.6217; *P* < 0.05) (Figure 3) and in normotensive subjects (*r* = 0.5886; *P* < 0.05) (Figure 6). More so positive correlation of systolic and diastolic blood pressure of normotensive subjects was observed across gender distribution (Figure 8) but negative correlation between systolic blood pressure and male hypertensive subjects (Figure 7). There was a statistical relationship between PT and APTT and age of hypertensive patients (*P* < 0.05) (Table 3). However there was no statistical relationship between PTT and APTT across ages of normotensive patients (Table 4).

## 4. Discussion

Haemostatic system is directly involved in the atherosclerotic process. Hypertension is an atherosclerotic risk factor causing endothelial dysfunction; therefore, endothelial damage, platelets hyperactivation, and drugs are involved in the coagulation and fibrinolytic system [29, 30]. Prothrombin time and activated partial thromboplastin time are important clinical parameters for assessing extrinsic and intrinsic factors/pathways of the coagulation system [30].

The findings of this study show significant increases in the prothrombin time and activated partial thromboplastin time of hypertensive patients when compared to those of the normotensives (control). This increase could be due to endothelial damage as a result of atherosclerosis caused by hypertension on these patients (*P* < 0.05) [31].

The prothrombin time and activated partial thromboplastin time of hypertensive patients increase with increase in duration of hypertension which may be due to prolonged endothelial wall effect as this will lead to sustained release of vasoactive substances that interfere coagulation cascades, and these indices eventually decrease as the antihypertensive therapy continues, even lower than before they started treatment; this finding is in consonance with Lee's work and that of Mirsaiedi et al. [32, 33].

There was a positive correlation between systolic pressure and activated partial thromboplastin time in hypertensive patients which could be due to the risk factors associated with hypertension, for example, atherosclerosis, endothelial dysfunction and antihypertensive drugs [34, 35].

There was a negative correlation between diastolic pressure and duration of illness and a probable explanation to this could be that reduced exposure of the vascular walls to pressure in those with lower duration will give a higher pressure than those who have higher exposure to this tension because their blood vessels might be weaker and cannot exert resistance or pressure against blood flow when compared to those with lower duration [35–38].

Positive correlation of systolic and diastolic blood pressure of normotensive subjects was observed across gender distribution, but negative correlation between systolic blood pressure in male hypertensive subjects; these findings are in conformity with previous studies [38–41]. There was a statistical relationship between PT and APTT and age distribution of hypertensive patients (*P* < 0.05); this supports the findings of Akputuzor et al. and Mirsaiedi et al. [33, 42]. Prolonged PT and APTT were common with elderly hypertensive subjects; one could speculate that this phenomenon is due to diminished prostacyclin synthesis and/or release by the endothelial cells during old age.

Due to the fact that there was significant increase in prothrombin time and activated partial thromboplastin time of hypertensive patients when compared with normotensive (control) subjects, assessment of these parameters may serve as prognostic indices for evaluating hypertensive patients in whom there was clinical evidence of hemostatic abnormality and guide for antihypertensive therapy. However, to have complete understanding of hemostatic activities in hypertension, it is recommended to conduct D-dimer, platelet factors, and protein assays.

## Figures and Tables

**Table 1:** Mean systolic pressure, diastolic pressure, prothrombin time test (PTT), and partial thromboplastin time test kaolin (PTTK) of hypertensive patients (test) and normotensive subjects (control) of the University of Calabar Teaching Hospital.

Parameters	Hypertensive patients (*n*_1_ = 42)	Normotensive subjects (*n*_2_ = 30)	*P* value
Systolic blood pressure (mmHg)	157.02 ± 18.77	117.66 ± 11.65	*P* < 0.05

Diastolic blood pressure (mmHg)	95.11 ± 12.99	79.16 ± 10.99	*P* < 0.05

PTT (seconds)	14.45 ± 1.96	13.60 ± 1.19	*P* < 0.05

PTTK (seconds)	35.42 ± 5.04	32.56 ± 3.23	*P* < 0.05

Student's *t*-test analysis.

*P* < 0.05 is significant.

*P* < 0.05 is not significant.

*n*_1_ = number of hypertensive patients.

*n*_2_ = number of normotensive subjects.

**Table 2:** Comparison of the prothrombin time test (PTT) and partial thromboplastin time test kaolin (PTTK) based on duration of illness of hypertensive patients attending the University of Calabar Teaching Hospital.

Parameters	Groups			*P* value
	≤3 years (*n*_1_ = 28)	4–7 years (*n*_2_ = 8)	≥8 years (*n*_3_ = 6)	
PTT (seconds)	14.28 ± 1.80	15.00 ± 2.67	14.50 ± 1.87	*P* > 0.05
PTTK (seconds)	34.89 ± 4.57	37.37 ± 6.07	35.33 ± 6.05	*P* > 0.05

Analysis of variance.

*P* < 0.05 is significant.

*P* < 0.05 is not significant.

*n*_1_ = number of hypertensive patients with duration of illness ≤3 years.

*n*_2_ = number of hypertensive patients with duration of illness 4–7 years.

*n*_3_ = number of hypertensive patients with duration of illness ≥8 years.

**Table 3:** Comparison of the prothrombin time test (PTT) and partial thromboplastin time test kaolin (PTTK) across ages of hypertensive patients attending the University of Calabar Teaching Hospital.

Parameters	Age range			*P* value
	≤35 years (*n*_1_ = 5)	36–50 years (*n*_2_ = 14)	≥51 years (*n*_3_ = 23)	
PTT (seconds)	13.80 ± 1.92	15.00 ± 1.96	14.26 ± 1.98	*P* < 0.05
PTTK (seconds)	32.60 ± 4.39	38.29 ± 3.60	34.3 ± 5.29	*P* < 0.05

Analysis of variance.

*P* < 0.05 is significant.

*P* < 0.05 is not significant.

*n*_1_ = number of hypertensive patients with age range ≤35 years.

*n*_2_ = number of hypertensive patients with age range 36–50 years.

*n*_3_ = number of hypertensive patients with age range ≥51 years.

**Table 4:** Comparison of the prothrombin time test (PTT) and partial thromboplastin time test kaolin (PTTK) across ages of normotensive patients attending the University of Calabar Teaching Hospital.

Parameters	Age range			*P* value
	≤35 years (*n*_1_ = 16)	32–50 years (*n*_2_ = 7)	≥51 years (*n*_3_ = 7)	
PTT (seconds)	13.75 ± 1.24	13.00 ± 1.154	13.86 ± 1.069	*P* > 0.05
PTTK (seconds)	29.19 ± 8.06	27.14 ± 10.75	32.71 ± 4.46	*P* > 0.05

Analysis of variance.

*P* < 0.05 is significant.

*P* < 0.05 is not significant.

*n*_1_ = number of normotensive patients with age range ≤35 years.

*n*_2_ = number of normotensive patients with age range 36–50 years.

*n*_3_ = number of normotensive patients with age range ≥51 years.
